# First Case of Dendriform Pulmonary Ossification in Bahrain

**DOI:** 10.7759/cureus.12904

**Published:** 2021-01-25

**Authors:** Naser Naser, Sayed Mohammed Jawad Alwedaie, Husain Kadhem

**Affiliations:** 1 Internal Medicine, Salmaniya Medical Complex, Manama, BHR

**Keywords:** high-resolution ct scan, pulmonary ossification, reticulonodular opacities, asymptomatic presentation

## Abstract

Dendriform pulmonary ossification (DPO) is a rare interstitial lung disease characterised by the presence of mature bone with marrow elements in the lung parenchyma with typical radiologic findings of diffuse and numerous calcified nodules. In this case study, we are presenting a case of asymptomatic primary DPO discovered during routine screening chest X-ray. To our best knowledge, this is the first case of DPO reported in Bahrain.

## Introduction

Pulmonary ossification is a rare disease condition of the lung in which progressive metaplastic ossification occurs in the interstitial space. Because most of the patients having this condition remain asymptomatic throughout their lives, the diagnosis is usually made on post mortem autopsy or surgical biopsy specimens [1]. The two types of widely known pulmonary ossifications are nodular type and dendriform type. Here we describe the history and diagnostic findings of a patient whose abnormal chest X-ray resulted in his referral to the respiratory clinic in our tertiary care hospital.

## Case presentation

**Figure 1:**
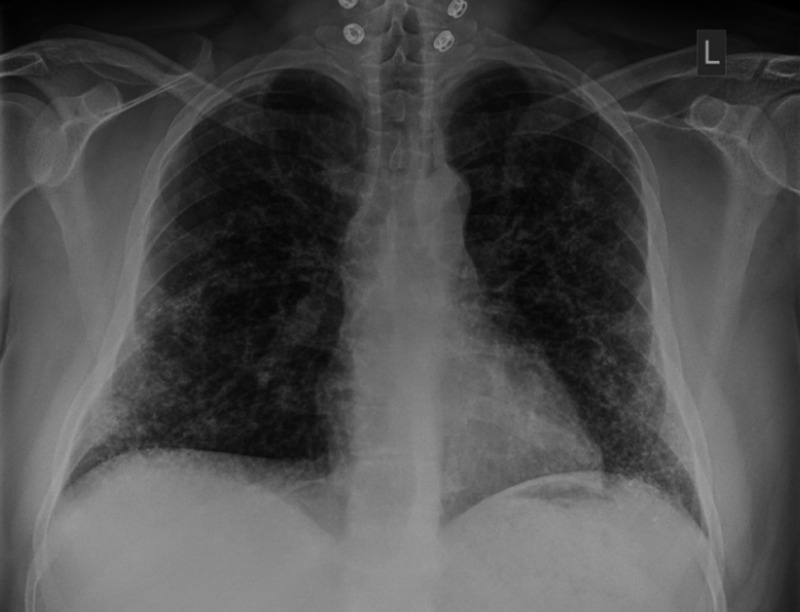
Erect chest X-ray (posteroanterior view) In this chest X-ray, we can see numerous tiny dense nodules extensively distributed in both lungs especially in the lower zones and in a branching (dendriform) pattern. Reticulations from thickening of the interlobular septa can be appreciated as well.

A 35-year-old Bahraini male patient, non-smoker, with no previous medical illness, referred to the respiratory clinic for evaluation of abnormal chest X-ray which was showing bilateral reticulonodular opacities distributed all over lung zones, more prominently in the lower lobes (Figure 1).

The patient denied any history of fever, cough, shortness of breath, weight loss, arthralgia or skin rashes. The patient gave history of past exposure to birds (pigeon and parrot), but this exposure has stopped for 10 years. There was not any significant occupational exposure as the patient works in an office.

His chest examination was unremarkable and no crackles or wheezes were heard upon auscultation. No digital clubbing was noticed in hand exam. His laboratory test yielded normal erythrocyte sedimentation rate (ESR), normal kidney function and electrolytes including calcium. Auto immune markers including anti-nuclear antibody (ANA), complement component three (C3), complement component four (C4) and rheumatoid arthritis (RA) latex, were all negative. Pulmonary function test (PFT) was conducted and demonstrated modest restrictive pattern. The recorded values for forced expiratory volume in the first one second (FEV-1), forced vital capacity (FVC), total lung capacity (TLC) and the FEV-1/FVC ratio can be seen in Table 1.

**Table 1:** Pulmonary function test (PFT) results FEV-1: Forced expiratory volume in the first one second; FVC: Forced vital capacity; TLC: Total lung capacity.

Pulmonary function test (PFT)	Results at the time of diagnosis	Results two years after the diagnosis
FEV-1/FVC	84%	71%
FEV-1	67%	66%
FVC	65%	77%
TLC	64%	Not done

**Figure 2:**
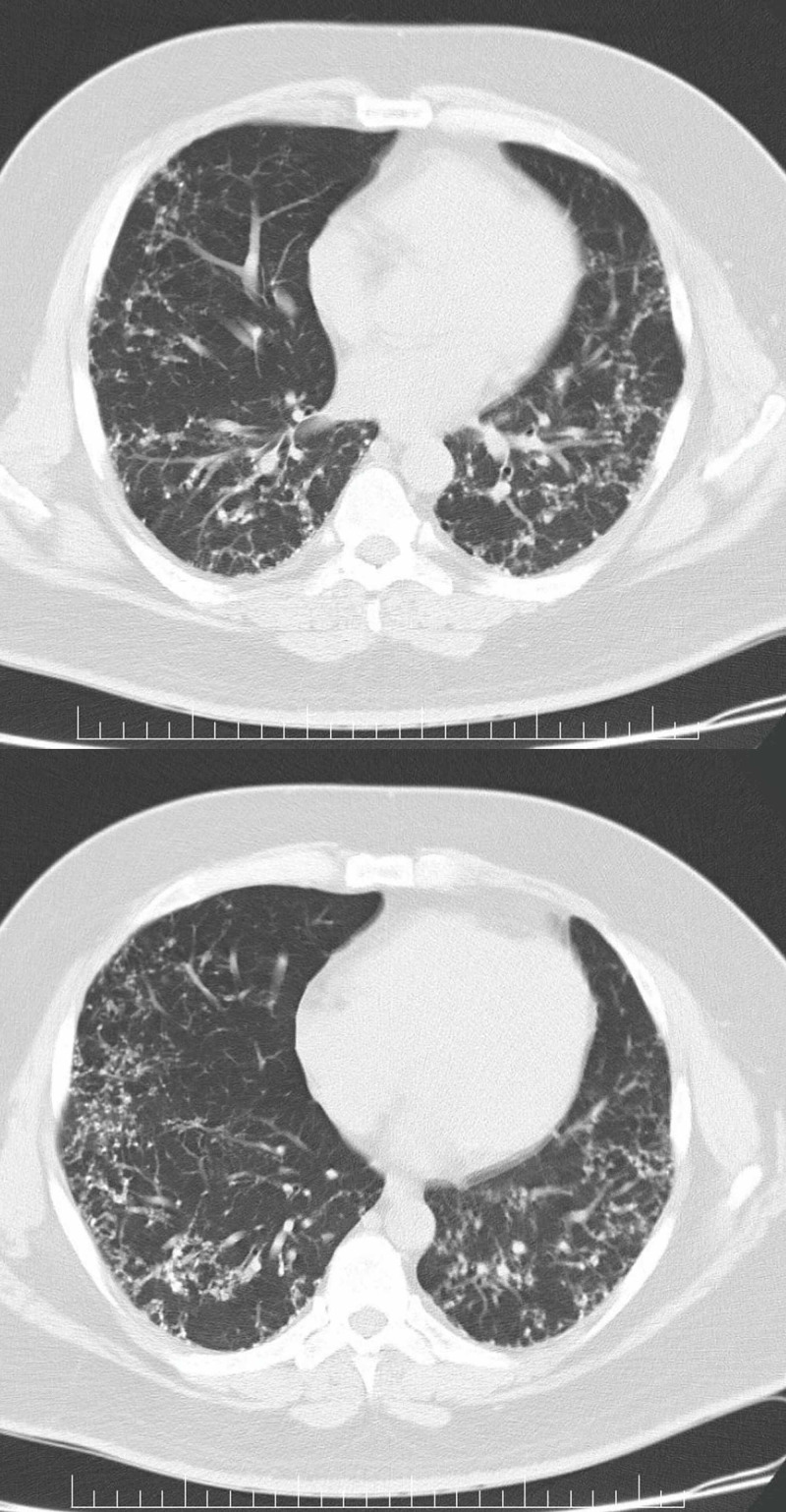
High-resolution computed tomography (CT) scan of the chest (at the heart level) The above CT images demonstrate nodular thickening of the interlobular septa more prominent in the sub-pleural regions in a lattice-like appearance. Also, dense nodules can be observed along the interlobular septa and in the sub-pleural interstitium.

High-resolution computed tomography (CT) scan of the chest was obtained. There was nodular thickening of the interlobular septa more prominent in the sub-pleural region. Also, dense nodules were observed along the interlobular septa and in the sub-pleural interstitium. No consolidation, ground glass opacity or pleural effusion was appreciated (Figure 2).

**Figure 3:**
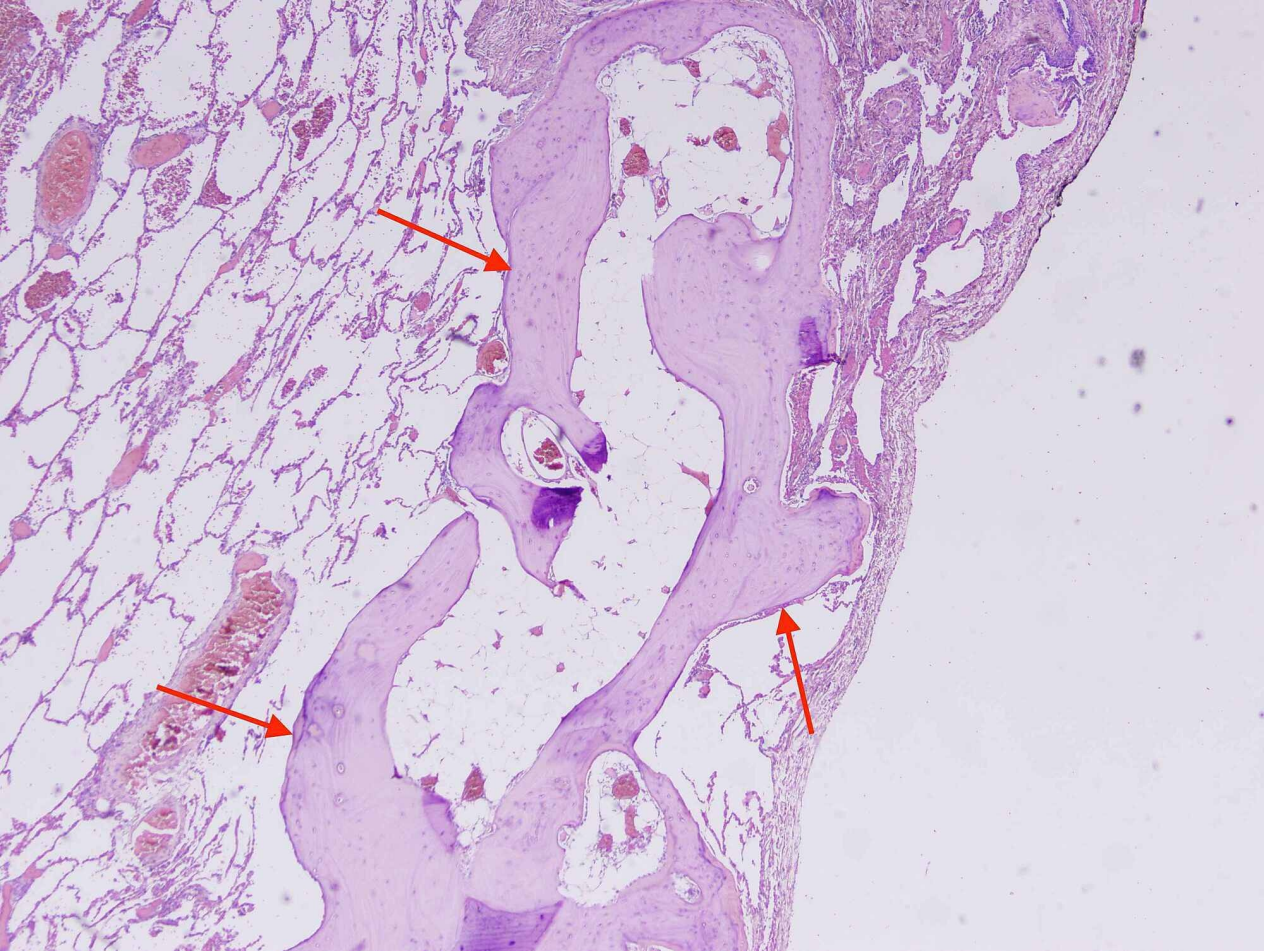
Histological section of lung parenchyma (H&E) This slide shows lung parenchyma with mature bone formation in the interstitial pulmonary spaces (red arrows). H&E: hematoxylin and eosin

Further workup revealed negative avian precipitants. Test for angiotensin converting enzyme (ACE) levels showed a value within normal range. Subsequently, the patient was subjected to bronchoscopy procedure with transbronchial biopsy, but it proved to be inconclusive to help forming a final diagnosis, as it was showing lymphoplasmacytic cell infiltration with dystrophic calcification. The patient underwent surgical lung biopsy procedure. Microscopy of the obtained tissue illustrated lung parenchyma with mature bone formation in the interstitial pulmonary spaces. Elements of fatty marrow were observed in some of these bones, however, no granuloma, atypical or malignant cells were noticed (Figure 3).

In a multidisciplinary team discussion between respiratory, radiology and pathology departments, the final diagnosis was decided to be dendriform pulmonary ossification (DPO). The patient was discussed regarding his new clinical diagnosis. As he was asymptomatic, he was offered a regular follow-up in the respiratory clinic plus monitoring of his lung function. The patient has been on regular follow-up for two years in the outpatient clinic and his last PFT results (two years after the diagnosis) showed a stable lung function. The last PFT results can be seen in Table 1.

## Discussion

Pulmonary ossification can be categorised according to two histological findings: nodular (granular) and dendriform. The nodular type is more common than the dendriform type (the present case is dendriform type). Most of the cases reported occurred in lower lung zones (which is consistent with the present case). Histologic examination of lung biopsies taken from patients with dendriform pulmonary ossification (DPO), demonstrates calcification in a collagen matrix within the lung parenchyma [2-4]. The nodular type has the characteristic lobulated bone nodules within alveolar spaces without marrow or fat elements [1]. In DPO, branching bony tissue can be seen with marrow elements which mainly affects the interstitium of the alveoli which can extend through the alveolar septum and less likely alveolar spaces [3]. Histologically, dendriform pattern has been identified in several other conditions including amyloidosis, asbestosis, cystic fibrosis and busulfan therapy [1].

From radiological point of view, computed tomography (CT) scans are believed to be superior to chest radiographs for diagnosis of pulmonary ossifications [1]. Chest X-rays usually illustrate bilateral nonspecific reticulonodular opacities, affecting the lower lobes predominantly. The possible differential diagnosis that can be made from the chest X-ray includes bronchiectasis, pulmonary fibrosis and lymphatic spread of a tumour [1]. CT scans can detect calcium deposits within the affected lung and more specifically, high-resolution CT scans (HRCT) can accurately demonstrate branching lines of calcifications which are fairly the characteristics of DPO. HRCT scans can differentiate DPO from other causes of diffuse calcifications like pulmonary alveolar microlithiasis which typically presents in the shape of calcified micronodules [1].

Majority of pulmonary ossification cases are diagnosed in post-mortem exam, largely due to lack of clinical or radiological findings. A study has found that male to female ratio is usually 6:1 and that age of 65 is the mean age in which diagnosis is made [5]. Our patient was notably young at the time of his diagnosis.

There are several hypotheses explained to be the cause of pulmonary ossification. The nodular type is found to be associated with passive congestions secondary to mitral stenosis and chronic heart failure [1]. The dendriform type could be an idiopathic condition or could be due to a pre-existing lung condition like chronic obstructive pulmonary disease (COPD), acute respiratory distress syndrome (ARDS), organising pneumonia, asbestosis and medication-induced lung injury [3, 6-10]. Our patient did not report any previous history of lung disease, so in his case, DPO is expected to be idiopathic.

Interaction between pulmonary fibroblasts and macrophages is thought to be the leading pathway to DPO which can be induced by cytokines, enzymes and free radicals [11, 12].

Clinically speaking, majority of patients are either asymptomatic at the time of presentation or they might exhibit mild cough or dyspnoea [6]. Pneumothorax is a rare complication of DPO according to the available reports [2, 12-17]. Our case was asymptomatic at the time of presentation and remained so at his last visit to our respiratory clinic.

There is no specific treatment strategy to manage patients with DPO and the available studies focus on symptomatic treatment and relieving complications [18]. Lung transplantation remains for advanced stages of the disease, when patient’s condition gets complicated by respiratory failure [19].

## Conclusions

In this case report, we presented a case of DPO, a rare interstitial lung disease which mostly presents asymptomatically. It could be either idiopathic or the result of a chronic lung disease. By choosing appropriate diagnostic tools, clinicians will be able to form accurate diagnosis and thus formulate a proper management option. The available studies focus on symptomatic relief for DPO and lung transplant is the treatment of last resort.
